# Relation Between *V˙E/V˙CO2* Slope and Maximum Phonation Time in Chronic Heart Failure Patients

**DOI:** 10.1097/01.md.0000464206.70920.58

**Published:** 2015-01-16

In the article “Relation Between *V˙E/V˙CO2* Slope and Maximum Phonation Time in Chronic Heart Failure Patients”,^1^ which appeared in Volume 93, Issue 29 of *Medicine*, the acting editor's name, Salvatore Patanè, was incorrectly spelled as Salvatore Patane. Salvatore Patanè's name also incorrectly appeared online as an author of the paper. He was the editor of the paper, but not an author.

## References

[1] Izawa KP Watanabe S Brubaker PH Relation Between *V˙E/V˙CO2* Slope and Maximum Phonation Time in Chronic Heart Failure Patients. *Medicine* 93 (29):e306. 25546676 10.1097/MD.0000000000000306 PMC4602617

